# Secretome Analysis of Rabbit and Human Mesenchymal Stem and Endothelial Progenitor Cells: A Comparative Study

**DOI:** 10.3390/ijms222212283

**Published:** 2021-11-13

**Authors:** Jaromír Vašíček, Andrej Baláži, Mária Tirpáková, Andrea Svoradová, Ľubomír Ondruška, Vladimír Parkányi, Peter Chrenek

**Affiliations:** 1 Institute of Farm Animal Genetics and Reproduction, NPPC, Research Institute for Animal Production in Nitra, Hlohovecká 2, 951 41 Lužianky, Slovakia; andrej.balazi@nppc.sk (A.B.); andrea.svoradova@nppc.sk (A.S.); lubomir.ondruska@nppc.sk (Ľ.O.); vladimir.parkanyi@nppc.sk (V.P.); 2 Institute of Biotechnology, Faculty of Biotechnology and Food Science, Slovak University of Agriculture in Nitra, Tr. A. Hlinku 2, 949 76 Nitra, Slovakia; maria.tirpakova@uniag.sk; 3 AgroBioTech Research Center, Slovak University of Agriculture in Nitra, Tr. A. Hlinku 2, 949 76 Nitra, Slovakia; 4 Department of Morphology, Physiology and Animal Genetics, Faculty of Agri Sciences, Mendel University in Brno, Zemědělská 1/1665, 613 00 Brno, Czech Republic

**Keywords:** rabbit, adipose tissue, MSCs, EPCs, cytokine array

## Abstract

Human adipose tissue-derived mesenchymal stem cells (AT-MSCs) have been studied several years for their immunomodulatory effect through the paracrine mechanism and cytokine secretion. In combination with endothelial progenitor cells (EPCs), MSCs have great therapeutical potential for the repair of endothelium and wound healing. However, little is known about the cytokine profile of rabbit AT-MSCs or even EPCs. The aim of this study was to analyze the secretomes of these rabbit stem/progenitor cells. A large-scale human cytokine array (up to 80 cytokines) was used to identify and compare cytokines secreted into conditioned media of human and rabbit AT-MSCs as well as HUVECs and rabbit EPCs. Few cytokines were highly expressed by human AT-MSCs (TIMP-2, TIMP-1), HUVECs (MCP-1, TIMP-2, GRO, Angiogenin, IL-8, TIMP-1), or by rabbit EPCs (TIMP-2). Several cytokines have moderate expression by human (MCP-1, GRO, Angiogenin, TGF-β 2, IL-8, LIF, IL-6, Osteopontin, Osteoprotegerin) and rabbit AT-MSCs (TIMP-2, TGF-β 2, LIF, Osteopontin, IL-8, IL-5, IL-3) or by HUVECs (IL-6, MIF, TGF-β 2, GCP-2, IGFBP-2, Osteoprotegerin, EGF, LIF, PDGF-BB, MCP-3, Osteopontin, Leptin, IL-5, ENA-78, TNF-β) and rabbit EPCs (TGF-β 2, Osteopontin, GRO, LIF, IL-8, IL-5, IL-3). In conclusion, the proposed method seems to be useful for the secretome analysis of rabbit stem/progenitor cells.

## 1. Introduction

For almost the last two decades, researchers have focused on the study of mesenchymal stem cells (MSCs) due to their specific biological features. This specific cell type can be presently obtained from various human as well as rabbit biological sources, most often from bone marrow, amniotic fluid, or others [1,2,3,4]. However, recently, adipose tissue became preferred for the isolation of MSCs as a more feasible biological source from human or rabbit [1,5]. Adipose tissue-derived MSCs (AT-MSCs) are very interesting for the cellular therapy, as they show an immuno-regulatory ability via cytokine secretion or the expression of cytokine receptors [6]. Moreover, their immunomodulatory effect is similar to that of MSCs derived from bone marrow [7], thus indicating a greater stem cell-based immune-therapeutical potential of AT-MSCs. In addition, MSCs in general might control neovascularization and secret different angiogenic, anti-apoptotic, immunosuppressive, or proliferation-stimulating factors [8].

Endothelial progenitor cells (EPCs) are crucial for the vascular repair after injury and in addition maintain the integrity of the endothelium in general. EPCs, originally resided in bone marrow, are circulating in the peripheral blood and when in need migrate to the site of injury and differentiate into mature cells of the endothelium. Moreover, these immature endothelial cells possess features of hematopoietic stem and/or progenitor cells [9]. According to existing studies, the paracrine mechanism of EPCs and MSCs may have a beneficial effect on the repair of the endothelium through the growth factors secreted by both cell types [10,11]. These features make them both interesting for stem cell therapy, tissue engineering, and regenerative medicine. For such studies, rabbit stem and progenitor cells became also a favorable choice [2,4,5,12].

While the cytokine profile of human AT-MSCs has been examined in several studies [13,14,15,16], the secretome of rabbit AT-MSCs is practically unknown. Moreover, only a few studies are available in the literature, which focused on the detection of selected cytokines secreted by rabbit MSCs derived from bone marrow [17,18]. The same goes also for rabbit EPCs, since there is only one known study focusing on the cytokines secreted by those rabbit cells [19]. Thus, a large-scale analysis of the cytokine profile typical for rabbit stem/progenitor cells might be of great interest in order to improve the basic knowledge about these rabbit cells as well as for their further therapeutic use.

Therefore, in this study, we focused on the assessment of cytokines secreted by rabbit AT-MSCs and EPCs. As far as we know, this is the first study revealing the secretome of rabbit AT-MSCs. Moreover, we compare the secretome of rabbit AT-MSCs with human AT-MSCs. The secretomes of rabbit EPCs were compared to cytokines secreted by human umbilical vein endothelial cells (HUVECs), as this cell line is commonly used for in vitro angiogenic assays.

## 2. Results

### 2.1. Full Cytokine Profile of Different Human and Rabbit Cells

**Figure 1:**
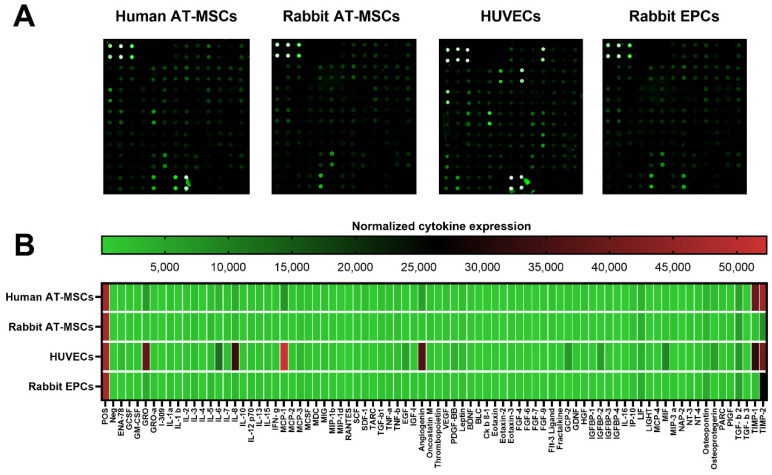
Expression of cytokines by human and rabbit stem/progenitor cells. Representative images of fluorescent cytokine antibody array for analyses of 80 cytokines in human and rabbit samples (**A**). Heat map showed a normalized expression of all analyzed cytokines in different human and rabbit samples (**B**). AT-MSCs—adipose tissue-derived mesenchymal stem cells; HUVECs—human umbilical vein endothelial cells; EPCs—endothelial progenitor cells.

The conditioned media from human and rabbit AT-MSCs, HUVECs, and rabbit EPCs were collected after 24 h of culture. All samples, human and rabbit, were analyzed using human cytokine antibody array, which allows analyzing up to 80 different cytokines in liquid samples (Figure 1A). As shown in the heat map (Figure 1B), only a few cytokines were highly expressed by human AT-MSCs (TIMP-2, TIMP-1), HUVECs (MCP-1, TIMP-2, GRO, Angiogenin, IL-8, TIMP-1), or by rabbit EPCs (TIMP-2). Any of the cytokines were highly expressed by rabbit AT-MSCs (Table 1). On the other hand, several cytokines have moderate expression by human (MCP-1, GRO, Angiogenin, TGF-β 2, IL-8, LIF, IL-6, Osteopontin, Osteoprotegerin) and rabbit AT-MSCs (TIMP-2, TGF-β 2, LIF, Osteopontin, IL-8, IL-5, IL-3) or by HUVECs (IL-6, MIF, TGF-β 2, GCP-2, IGFBP-2, Osteoprotegerin, EGF, LIF, PDGF-BB, MCP-3, Osteopontin, Leptin, IL-5, ENA-78, TNF-β) and rabbit EPCs (TGF-β 2, Osteopontin, GRO, LIF, IL-8, IL-5, IL-3). The majority of the analyzed cytokines have lower or weak expression (Table 1).

### 2.2. Comparison of the Mostly Secreted Cytokines by Different Human and Rabbit Cells

**Figure 2:**
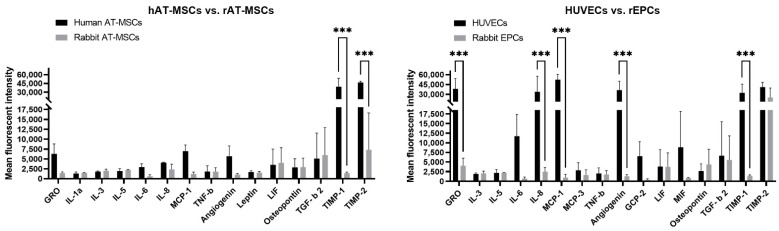
The mostly secreted cytokines compared according to the same cell type and different animal species. Ten cytokines with the highest expression in each cell type were chosen for the comparison. hAT-MSCs—human adipose tissue-derived mesenchymal stem cells; rAT-MSCs—rabbit adipose tissue-derived mesenchymal stem cells; HUVECs—human umbilical vein endothelial cells; rEPCs—rabbit endothelial progenitor cells. The data from three independent experiments are expressed as the means ± SD; ***—difference is statistically significant at *p* < 0.001.

To compare thoroughly the cytokine profile of analyzed cell samples, we chose ten mostly expressed cytokines by each cell type and animal species. As shown in Figure 2, human AT-MSCs secreted similar levels of cytokines (GRO, IL-1a, IL-3, IL-5, IL-6, IL-8, MCP-1, TNF-β, Angiogenin, Leptin, LIF, Osteopontin, and TGF-β 2) as rabbit AT-MSCs, except for significantly (*p* < 0.001) increased TIMP-1 and TIMP2 in human samples compared to rabbit samples. In case of endothelial cells, HUVECs secreted significantly (*p* < 0.001) higher levels of GRO, IL-8, MCP-1, Angiogenin, and TIMP-1 in comparison to rabbit EPCs. On the other hand, other mostly expressed cytokines (IL-3, IL-5, IL-6, MCP-3, TNF-β, CGP-2, LIF, MIF, Osteopontin, TGF-β 2, and TIMP2) did not differ between human and rabbit endothelial samples.

**Figure 3:**
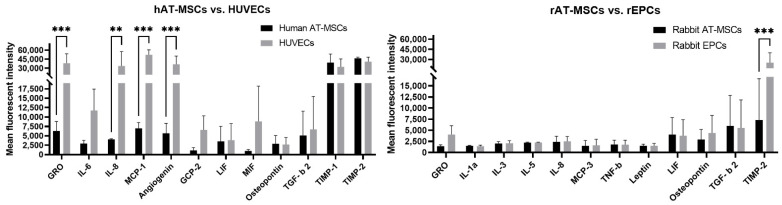
The mostly secreted cytokines compared according to the same animal species and different cell type. Ten cytokines with the highest expression in each cell type were chosen for the comparison. hAT-MSCs—human adipose tissue-derived mesenchymal stem cells; HUVECs—human umbilical vein endothelial cells; rAT-MSCs—rabbit adipose tissue-derived mesenchymal stem cells; rEPCs—rabbit endothelial progenitor cells. The data from three independent experiments are expressed as the means ± SD; **—difference is statistically significant at *p* < 0.01; ***—difference is statistically significant at *p* < 0.001.

Human cell types (hAT-MSCs and HUVECs) secreted similar levels of several cytokines (IL-6, GCP-2, LIF, MIF, Osteopontin, TGF-β 2, TIMP-1, and TIMP-2). However, HUVECs showed significantly increased expression of GRO, MCP-1, Angiogenin (*p* < 0.001), and IL-8 (*p* < 0.01) in comparison to hAT-MSCs (Figure 3). On the other hand, rabbit cell types (rAT-MSCs and rEPCs) did not significantly differ in the cytokine secretion profile (GRO, IL-1a, IL-3, IL-5, IL-8, MCP-3, TNF-β, Leptin, LIF, Osteopontin, and TGF-β 2). On the contrary, TIMP-2 cytokine was significantly (*p* < 0.001) oversecreted by rEPCs compared to rAT-MSCs.

The majority of the mostly secreted cytokines by all four studied cell types were pro-angiogenic and pro-inflammatory (Table 2), although some of them can act anti-inflammatory according to the specific circumstances such as TIMP-2, TGF-β 2, and LIF. Moreover, rAT-MSCs and rEPCs secreted also anti-inflammatory cytokine IL-5. Only few anti-angiogenic cytokines were found among the mostly secreted cytokines (TIMP-2 and also TIMP-1, except for rAT-MSCs and rEPCs, as shown in Table 2). All cell types also secreted higher levels of neuroregulatory cytokine LIF. Additionally, the majority of the expressed cytokines belongs to adipokines secreted by adipose tissue.

## 3. Discussion

Here, we used a human cytokine array to detect cytokines presented in the conditioned media of rabbit and human stem/progenitor cells. This array has already been successfully used in several human studies [8,20,21,22], but also in rabbit study [19] to assess the cytokine profile of rabbit EPCs. According to this array, all cell samples analyzed in our study similarly secreted at higher levels mainly pro-angiogenic and pro-inflammatory cytokines and few anti-angiogenic, anti-inflammatory, and neuroregulatory cytokines (Table 2). Moreover, a majority of the highly expressed cytokines might be also assigned to adipokines (cytokines, chemokines, and factors), including LIF (secreted only at certain stages of development [23]), which are secreted in adipose tissue and thus controlling adipogenesis. However, the secretion of typical adipokines such as leptin, IL-6, IL-8, or IL-1a etc. [24,25,26] was not significantly different when all analyzed cell samples were compared (Table 1, Figure 2 and Figure 3). It has been observed that human MSCs derived from bone marrow expressed various genes for angiogenic cytokines and thus affected the proliferation of HUVECs through the paracrine mechanism [11]. In a different study [8], the same type of human MSCs has also been reported to secrete classical factors associated with angiogenesis such as angiopoietin-2, bFGF, FGF-4, FGF-9, TIMP-1, TIMP-2, VEGF, and VEGF-D. In addition, MSCs from human cord blood secreted several similar cytokines such as FGF-4, IL-8, TIMP-1, TIMP-2, VEGF, etc. [27] as those from bone marrow. On the other hand, some of the cytokines expressed by cord blood MSCs (IL-6, GM-CSF, IGFBP-1, etc.) were not significantly expressed in bone marrow MSCs [8,27]. Moreover, a comparison of cytokines secreted by human MSCs derived from four different biological sources (cord blood, bone marrow, amnion, and decidua of the placenta) revealed several cytokines common to all MSCs types such as MIF, IL-8, Serpin E1, GROα, and IL-6 [28]. On the contrary, SDF-1 expressed only bone marrow MSCs, while MCP-1 was secreted by bone marrow and amnion MSCs and sICAM-1 was secreted by amnion and decidua MSCs [28]. The similar observation was reported for bone marrow and cord blood MSCs analyzed using protein membrane array consisting of 120 different cytokines. In this study, higher but slightly different concentrations of IL-6, IL-8, MCP-1, OPG, TIMP-2, and VEGF were noticed when comparing two different types of MSCs [29]. This suggests that MSCs from different sources may share the expression of some cytokines but may also secrete their own tissues-specific cytokines. Several previous studies analyzed the cytokines and other factors secreted by human AT-MSCs using ELISA or other protein assays [13,14,15,16]. Some of them assessed only a few selected cytokines and growth factors using the ELISA technique, which confirmed the secretion of IL-6, EGF, FGF, MCP-1, SDF-1, TGF-β 1, TGF-β 2, VCAM1, VEGF, and VEGF2 by these MSCs [13,14]. In the others, protein assays were used to evaluate the secretion of up to 40 cytokines. For example, higher concentrations of IL-1RA, IL-6, IL-8, G-CSF, GM-CSF, MCP-1, NGF, HGF, and VEGF were observed in the conditioned medium of human AT-MSCs, while a low secretion of other factors such as EGF, IL-10, IP-10, MIP-1a, MIP-1b, IL-7, IL-15, eotaxin, fractaline, IL-12p40, IL-12p70, and IL-17 was also detected [15]. In a different study, researchers demonstrated about 43 angiogenic factors (such as Angiogenin, bFGF, EGF, IGF-1, IL-6, MCP-1, MCP-3, PDGF-BB, RANTES, TGF-β, VEGF, etc.) to be secreted by human AT-MSCs [16]. In our large-scale protein array study, human AT-MSCs highly secreted angiogenic factors such as TIMP-1 and TIMP-2, and a moderate expression of other angiogenic, inflammatory, and neuroregulatory cytokines (e.g., Angiogenin, GRO, IL-6, IL-8, LIF, MCP-1, Osteopontin, Osteoprotegerin, TGF-β 2) was also observed (Table 1). Although the rest of the analyzed cytokines have low and/or very dim expression, our findings are similar to those in the above-mentioned human MSCs studies.

Although several studies about the cytokine profile of human AT-MSCs can be found in the literature, there is a lack of or even no information about cytokines secreted by rabbit AT-MSCs. In addition, only a few studies are available that discussed the secretion of selected cytokines analyzed by ELISA in rabbit bone marrow-derived MSCs [17,18]. One of them detected a higher secretion of HGF, moderate secretion of IGF-1 and bFGF, and low concentration of VEGF after 24 h of incubation. Interestingly, the concentration of the evaluated cytokines increased almost two-fold after 48 h of incubation [17]. In the second study, IL-6 was not observed within the detection limit, whereas LIF was highly secreted by rabbit bone marrow MSCs [18]. These findings are in agreement with our results for rabbit AT-MSCs, as we found a low secretion of IL-6 and VEGF after 24 h of incubation. However, a moderate secretion of angiogenic, inflammatory, and neuroregulatory cytokines (e.g., IL-3, IL-5, IL-8, LIF, Osteopontin, TIMP-2, TGF-β 2) was also observed (Table 1). Moreover, the secretion profile of rabbit cells was similar to human AT-MSCs, although TIMP-1 and TIMP-2 were overexpressed in human cells in comparison to rabbit MSCs (Figure 2). A study comparing rat bone marrow MSCs and AT-MSCs revealed a higher secretion of HGF, VEGF, IL-6, and PAI-1 in rat AT-MSCs in comparison to bone marrow cells. On the other hand, a lower expression of SDF-1α and similar expression of adrenomedullin, TNF-α, and leptin was found in rat AT-MSCs [30]. This supports the finding noticed in the above-mentioned human studies that the secretion profile of MSCs can differ according to the tissue of their origin.

Here, we compared the cytokine profile of HUVECs and rabbit EPCs (Table 1, Figure 2). Both endothelial cell cultures secreted angiogenic, inflammatory, and neuroregulatory factors (Table 2). However, significantly higher levels of GRO, IL-8, MCP-1, Angiogenin, and TIMP-1, although not TIMP-2, were detected in HUVECs compared to rabbit EPCs (Figure 2). Moreover, HUVECs expressed also significantly higher levels of some angiogenic cytokines (GRO, IL-8, MCP-1, and Angiogenin) than human AT-MSCs (Figure 3), which was probably due to the angiogenic properties of HUVECs. On the contrary, the ELISA method detected significantly lower levels of VEGF, bFGF, IL-6, PlGF, and MCP-1 in HUVECs when compared to human bone marrow MSCs [11]. In the other study, HUVECs analyzed by ELISA secreted significantly lower VEGF, SDF-1, and IGF-1 as well as HGF (although not significantly) in comparison to human EPCs [31]. On the other hand, mRNA analysis of human EPCs observed a higher expression of wound healing-related cytokines and growth factors such as PDGF-α, PDGF-β, and KGF rather than the expression of angiogenic factors (bFGF, TGF-β 1, TGF-β 2, or VEGF), although their protein expression was not analyzed [32]. Lower levels of VEGF and high levels of IL-8 were detected using ELISA in conditioned media of human EPCs by different research groups [33,34,35]. In our study, VEGF was secreted in lower amounts by both HUVECs and rabbit EPCs (Table 1), whereas IL-8 was oversecreted by HUVECs against rabbit EPCs (Figure 2). Interestingly, according to He and colleagues [19], early rabbit EPCs secreted mainly pro-angiogenic factors (EGF, FGF-4, FGF-9, HGF, IL-8, MCSF, MIF, NAP-2, Oncostantin M, Osteoprotegerin, PlGF, TGF-β 2, TPO, and VEGF), anti-angiogenic (IFN-g and TIMP-1), and neuroregulatory cytokines (BDNF and LIF). Although we used a similar cytokine array in our study, we cannot compare the levels of secreted cytokines by rabbit EPCs, as these data are not available in the above-mentioned study. Nevertheless, the majority of the mentioned cytokines was also observed in this study for rabbit EPCs with higher or lower expression (Table 1). Furthermore, slight differences in the cytokine detection can result from the different detection system used (fluorescence versus chemiluminescence detection) and/or the evaluation method. It should be noticed that rabbit EPCs showed a similar secretion profile to rabbit AT-MSCs, except for TIMP-2, which was overexpressed by rabbit EPCs (Figure 3). On the other hand, some significant differences were observed in the secretion of cytokines when comparing human and rabbit AT-MSCs or HUVECs and rabbit EPCs. These differences might be due to the different specificity and/or cross-reactivity of antibodies involved in the used protein array for rabbit cytokines. The major advantages of this method are its high sensitivity and specificity, low sample volume (10–100 µL per array), and compatibility with most sample types such as cell supernatants, cell or tissue lysate, plasma and serum, etc. with minimal sample processing. At present, up to 2000 human proteins and up to 800 murine proteins can be detected by such protein array according to the manufacturer. On the other hand, up to 10,000 proteins can be identified using mass spectroscopy [36,37] independently of the analyzed animal sample. In fact, the main limitation of the protein array is the availability of antibodies specific against analyzed proteins, actually cytokines. The other expected limitation of such arrays can be the degree of antibody cross-reactivity [38] and thus the ability of antibody to recognize specific antigens (cytokine) of different animal species, as was mentioned above. The last major limitation of this method is semi-quantitative analysis, which allows detecting only relative but not absolute expression levels of the analyzed cytokines. Nevertheless, the proposed method seems to be useful for the secretome analysis of rabbit stem/progenitor cells.

In general, conditioned media (CM) from MSCs may enhance the in vitro migration and proliferation of fibroblasts and thus accelerate the process of healing [39,40]. Moreover, it was reported that CM from human EPCs may have angiogenic and cytoprotective effects against the ischemic insults of brain microvascular endothelial cells [33]. Therefore, it should be of great interest to study thoroughly the CM of rabbit stem/progenitor cells in order to explore their therapeutic potential, e.g., in veterinary medicine or as animal models for the treatment of human disorders.

## 4. Materials and Methods

### 4.1. Animals

In this study, young (3–8 months old) and clinically healthy rabbits (*n* = 6) of the New Zealand White (NZW) line were used. The animals were reared as described previously [4]. The treatment of the animals was approved by the Ministry of Agriculture and Rural Development of the Slovak Republic no. SK U 18016 in accordance with the ethical guidelines presented in Slovak Animal Protection Regulation (RD 377/12), which conforms to the Code of Ethics of the EU Directive 2010/63/EU for animal experiments.

### 4.2. Collection of Biological Material and Isolation of Rabbit Primary Cells

Peripheral blood and subcutaneous fat were harvested from humanely sacrificed rabbits as described in our previous studies [5,12]. Briefly, mononuclear cells from peripheral blood were isolated using density-gradient centrifugation and resuspended in endothelial basal (EBM-2) medium supplemented with recombinant growth factors (Lonza, Walkersville, MD, USA); fetal calf serum (Biowest, Riverside, MO, USA), and penicillin/streptomycin solution (Thermo Fisher Scientific, Waltham, MA, USA). Then, the cells were plated into culture flasks and cultured until the 3rd passage, as described previously [12], in order to obtain pure cell culture of rabbit endothelial progenitor cells (rEPCs).

Adipose tissue samples were enzymatically digested using collagenase type I (Sigma Aldrich, Gillingham, UK) and filtered in order to obtain single cell suspension. Then, the cells were resuspended in α-MEM culture medium (Gibco^TM^, Thermo Fisher Scientific, Waltham, MA, USA) supplemented with fetal bovine serum (Sigma Aldrich, Gillingham, UK) and penicillin/streptomycin solution (Thermo Fisher Scientific, Waltham, MA, USA). Cell suspensions were plated into culture flasks and cultured until the 3rd passage, as described previously [5] in order to obtain a pure cell culture of rabbit adipose tissue-derived mesenchymal stem cells (rAT-MSCs).

### 4.3. Culture of Human Cell Lines

Human cell lines were obtained from ATCC (Primary Umbilical Vein Endothelial Cells; Normal, Human (HUVECs) (ATCC^®^ PCS-100-010^TM^) and Adipose-Derived Mesenchymal Stem Cells; Normal, Human (hAT-MSCs) (ATCC^®^ PCS-500-011^TM^; ATCC, Manassas, VA, USA). Both frozen cell lines were thawed in a water bath and cultured in a proper culture medium with supplements (EBM-2 for HUVECs and α-MEM for hAT-MSCs) until the 3rd passage, as described previously [5,12].

### 4.4. Preparing and Analyses of Conditioned Media

All cell cultures (rabbit and human) in the 3rd passage were cultured using proper media with supplements as described above until the confluency of 60–70%. Then, cells were washed using the serum-free basal media once and incubated in serum-free basal media without growth factors for 24 h (conditioned media). All conditioned media were collected, aliquoted, and stored at −80 °C until the secretome analysis.

Samples of conditioned media from rabbit EPCs and AT-MSCs as well as from human HUVECs and AT-MSCs were analyzed using a RayBio^®^ Human Cytokine Antibody Array G-Series 5 (RayBiotech Life, Peachtree Corners, GA, USA). This array is designed for semi-quantitative analysis of 80 different human cytokines (Table 3) from liquid samples based on a glass chip that is highly sensitive to simultaneously detect multiple cytokine expression levels. The protocol recommended by manufacturer was used to analyze the cytokines expressed by individual samples. Briefly, cytokines presented in conditioned media specifically bound to spots on the chip and were detected by a cocktail of biotin-conjugated antibodies. After incubation of the chip with HiLyte Plus™ Fluor 555–conjugated streptavidin, the chip was scanned with an InnoScan 710 Microarray Scanner (Innopsys, Carbonne, France) in a “green” channel (excitation frequency = 532 nm). The semi-quantitative analysis of protein expression according to the fluorescent intensity of analyzed spots was done using the Mapix software (Innopsys, Carbonne, France). The data (fluorescent signals) obtained from three independent experiments for each cell type were normalized to internal positive controls present on each slide using the RayBio^®^ Analysis Tool (S02-AAH-CYT-G5; RayBiotech Life, Peachtree Corners, GA, USA) developed by the manufacturer of the used protein array.

### 4.5. Statistical Analysis

Normalized data obtained from the analyses of all cell cultures were evaluated cytokine by cytokine using GraphPad Prism version 9.2.0 for Windows (GraphPad Software, San Diego, CA, USA) with two-way ANOVA followed by Sidak’s test for multiple comparisons. Results are expressed as the mean ± SD. *p*-values at *p* < 0.05 were considered as statistically significant. The full array analysis of 80 cytokines from each cell type is presented as a heat map.

## 5. Conclusions

Here, we presented novel information about the possibility to analyze the secretome of rabbit mesenchymal stem cells and endothelial progenitor cells using a large-scale cytokine array designed for human samples. Moreover, similar cytokine secretion was observed for rabbit AT-MSCs and EPCs with only slight differences as for human cells. Some differences were found in the levels of secreted cytokines when comparing human and rabbit cells lines. Anyway, further studies are needed to describe the possible paracrine effect of rabbit AT-MSCs on the proliferation and function of rabbit EPCs. The changes in the cytokine profile of conditioned media obtained after 48 h of the cell culture or the presence of exosomes may be also examined in the future. Further studies may also focus on the co-culture of rabbit AT-MSCs themselves or their conditioned media with rabbit EPCs.

## Figures and Tables

**Table 1:** Overview of analyzed cytokines according to their normalized expression in different cell types.

Cell Type/Mean Fluorescent Intensity	Human AT-MSCs	Rabbit AT-MSCs	HUVECs	Rabbit EPCs
60,000–25,000	TIMP-2, TIMP-1	N.D.	MCP-1, TIMP-2, GRO, Angiogenin, IL-8, TIMP-1	TIMP-2
25,000–2000	MCP-1, GRO, Angiogenin, TGF-β 2, IL-8, LIF, IL-6, Osteopontin, Osteoprotegerin	TIMP-2, TGF-β 2, LIF, Osteopontin, IL-8, IL-5, IL-3	IL-6, MIF, TGF-β 2, GCP-2, IGFBP-2, Osteoprotegerin, EGF, LIF, PDGF-BB, MCP-3, Osteopontin, Leptin, IL-5, ENA-78, TNF-β	TGF-β 2, Osteopontin, GRO, LIF, IL-8, IL-5, IL-3
2000–200	IL-5, MCP-3, TNF-β, IL-3, SDF-1, Leptin, TGF-β 1, VEGF, IL-1a, IP-10, HGF, Oncostatin M, IL-7, IL-15, IGFBP-3, IL-13, TNF-α, Flt-3 Ligand, GCP-2, IL-12 p70, MIG, MCSF, IL-2, NT-4, MIF, SCF, PARC, GCSF, IFN-g, IGF-I, NT-3, GM-CSF, Eotaxin-3, MCP-2, IL-10, BDNF, MIP-1b, PlGF, EGF, Thrombopoietin, GRO-a, FGF-9, GDNF, FGF-6, MDC, ENA-78, FGF-4, PDGF-BB, MCP-4, MIP-3 a, IGFBP-1, IL-1 b, TARC, Ck b 8-1, IGFBP-2, LIGHT, RANTES, Eotaxin, FGF-7, BLC, Fractalkine, MIP-1d, I-309, Eotaxin-2, IGFBP-4	TNF-β, Leptin, IL-1a, MCP-3, GRO, IP-10, Oncostatin M, TIMP-1, TGF-β 1, IL-12 p70, NT-4, SDF-1, IL-7, IL-15, MIP-3 a, MCP-1, VEGF, PARC, TNF-α, MCSF, NT-3, IFN-g, Angiogenin, MIF, Eotaxin-3, Flt-3 Ligand, IGF-I, SCF, MDC, GRO-a, IL-2, IL-13, IGFBP-3, MIG, GCSF, BDNF, GDNF, MIP-1b, FGF-9, GM-CSF, PlGF, IL-10, MCP-2, FGF-6, Thrombopoietin, FGF-4, IGFBP-1, IL-6, EGF, PDGF-BB, MCP-4, HGF, TARC, ENA-78, IL-1 b, BLC, IGFBP-2, Eotaxin, RANTES, GCP-2, MIP-1d, IGFBP-4, Fractalkine, Eotaxin-2, IL-4, I-309, FGF-7, Ck b 8-1, LIGHT, Osteoprotegerin, NAP-2, IL-16	IL-3, IL-7, IP-10, MIG, IL-15, IL-1a, TNF-a, IL-12 p70, TGF-β 1, IL-13, HGF, Eotaxin-3, Oncostatin M, IL-2, VEGF, NT-4, PlGF, MCSF, SCF, IGFBP-3, GCSF, IFN-g, Flt-3 Ligand, GM-CSF, IL-10, MIP-1b, MCP-2, PARC, LIGHT, MDC, SDF-1, FGF-9, GRO-a, BLC, FGF-6, Fractalkine, GDNF, IGF-I, Thrombopoietin, Eotaxin, NT-3, FGF-4, MCP-4, Ck b 8-1, NAP-2, IL-1 b, TARC, MIP-1d, FGF-7, BDNF, Eotaxin-2, MIP-3 a, IGFBP-4, RANTES	TNF-β, MCP-3, Leptin, IL-1a, TIMP-1, SDF-1, IL-12 p70, TGF-β 1, Angiogenin, IP-10, Oncostatin M, PARC, EGF, IL-7, NT-4, IL-15, MCSF, Flt-3 Ligand, IGF-I, VEGF, TNF-α, IL-13, SCF, NT-3, IL-2, MIP-3 a, Eotaxin-3, MCP-1, IFN-g, MDC, GRO-a, GCSF, MIF, BDNF, PlGF, MIG, IGFBP-3, GM-CSF, MCP-2, Thrombopoietin, IL-10, MIP-1b, IL-6, FGF-6, FGF-9, GDNF, FGF-4, IGFBP-1, MCP-4, PDGF-BB, Ck b 8-1, HGF, TARC, IL-1 b, IGFBP-2, Eotaxin, BLC, IGFBP-4, Eotaxin-2, RANTES, GCP-2, LIGHT, Osteoprotegerin, FGF-7, MIP-1d, IL-4, ENA-78, Fractalkine, I-309, NAP-2
<200 (negative)	IL-16, IL-4, NAP-2, TGF-β 3	TGF-β 3	IGFBP-1, IL-4, I-309, IL-16, TGF-β 3	IL-16, TGF-β 3

Cytokines in the table cells are arranged according to their descending expression (mean fluorescent intensity); N.D.—not detected within the range specific for the table line.

**Table 2:** The mostly expressed cytokines by different cell types classified according to their biological function.

Cell Type/ Biological Function of Cytokine	Human AT-MSCs	Rabbit AT-MSCs	HUVECs	Rabbit EPCs
Pro-angiogenic	MCP-1, GRO, Angiogenin, TGF-β 2, IL-8, IL-6, Osteopontin	TGF-β 2, Osteopontin, IL-8, IL-5, IL-3, TNF-β, Leptin, IL-1a	MCP-1, GRO, Angiogenin, IL-8, IL-6, MIF, TGF-β 2, GCP-2	TGF-β 2, Osteopontin, GRO, IL-8, IL-5, IL-3, TNF-β, MCP-3
Anti-angiogenic	TIMP-2, TIMP-1	TIMP-2	TIMP-2, TIMP-1	TIMP-2
Pro-inflammatory	TIMP-1, MCP-1, GRO, Angiogenin, TGF-β 2 (according to circumstances), IL-8, LIF (according to circumstances), IL-6, Osteopontin	TGF-β 2 and LIF (according to circumstances), Osteopontin, IL-8, IL-3, TNF-β, Leptin, IL-1a	MCP-1, GRO, Angiogenin, IL-8, TIMP-1, IL-6, MIF, TGF-β 2 (according to circumstances), GCP-2	TGF-β 2 (according to circumstances), Osteopontin, GRO, LIF (according to circumstances), IL-8, IL-3, TNF-β, MCP-3
Anti-inflammatory	TIMP-2, TGF-β 2 and LIF (according to circumstances)	TIMP-2, TGF-β 2 and LIF (according to circumstances), IL-5	TIMP-2, TGF-β 2 (according to circumstances)	TIMP-2, TGF-β 2 and LIF (according to circumstances), IL-5
Neuroregulatory	LIF	LIF	LIF	LIF
Adipokines	TIMP-2, TIMP-1, MCP-1, GRO, Angiogenin, TGF-β 2, IL-8, LIF, IL-6, Osteopontin	TIMP-2, TGF-β 2, LIF, Osteopontin, IL-8, IL-5, Leptin, IL-1a	MCP-1, TIMP-2, GRO, Angiogenin, IL-8, TIMP-1, IL-6, MIF, TGF-β 2, GCP-2	TIMP-2, TGF-β 2, Osteopontin, GRO, LIF, IL-8, IL-5, MCP-3

**Table 3:** Complete list of analyzed cytokines in alphabetical order.

Human Cytokine Array G5				
Angiogenin	BDNF	BLC (CXCL13)	CK b 8-1 (CCL23)	EGF
ENA-78 (CXCL5)	Eotaxin (CCL11)	Eotaxin-2 (MPIF-2/CCL24)	Eotaxin-3 (CCL26)	FGF-4
FGF-6	FGF-7 (KGF)	FGF-9	Flt-3 Ligand	Fractalkine (CX3CL1)
GCP-2 (CXCL6)	GCSF	GDNF	GM-CSF	GRO-a (CXCL1)
GRO	HGF	I-309 (TCA-3/CCL1)	IFN-g	IGF-I
IGFBP-1	IGFBP-2	IGFBP-3	IGFBP-4	IL-1a (IL-1 F1)
IL-1 b (IL-1 F2)	IL-2	IL-3	IL-4	IL-5
IL-6	IL-7	IL-8 (CXCL8)	IL-10	IL-12 p40/p70
IL-13	IL-15	IL-16	IP-10 (CXCL10)	Leptin
LIF	LIGHT (TNFSF14)	MCP-1 (CCL2)	MCP-2 (CCL8)	MCP-3 (MARC/CCL7)
MCP-4 (CCL13)	MCSF	MDC (CCL22)	MIF	MIG (CXCL9)
MIP-1b (CCL4)	MIP-1d (CCL15)	MIP-3 a (CCL20)	NAP-2 (PPBP/CXCL7)	NT-3
NT-4	Oncostatin M	Osteopontin (SPP1)	Osteoprotegerin (TNFRSF11B)	PARC (CCL18)
PDGF-BB	PlGF	RANTES (CCL5)	SCF	SDF-1 alpha (CXCL12 alpha)
TARC (CCL17)	TGF-β 1	TGF-β 2	TGF-β 3	Thrombopoietin (TPO)
TIMP-1	TIMP-2	TNF-α	TNF-β (TNFSF1B)	VEGF

BDNF—Brain-derived neurotrophic factor; BLC—B lymphocyte chemoattractant; CK b 8-1 (CCL23)—C-C motif chemokine 23; EGF—Pro-epidermal growth factor; ENA-78—Epithelial-derived neutrophil-activating protein 78; FGF-4—Fibroblast growth factor 4; FGF-6—Fibroblast growth factor 6; FGF-7—Fibroblast growth factor 7; FGF-9—Fibroblast growth factor 9; Flt-3 Ligand—Fms-related tyrosine kinase 3 ligand; GCP-2—Granulocyte chemotactic protein 2; GCSF—Granulocyte colony-stimulating factor; GDNF—Glial cell line-derived neurotrophic factor; GM-CSF—Granulocyte–macrophage colony-stimulating factor; GRO-a—Growth-regulated alpha protein; GRO—Reacts with CXCL1, CXCL2, and CXCL3 (GRO alpha, beta, and gamma, respectively); HGF—Hepatocyte growth factor; I-309 (CCL1)—C-C motif chemokine 1; IFN-g—Interferon gamma; IGF-I—Insulin-like growth factor I; IGFBP-1—Insulin-like growth factor-binding protein 1; IGFBP-2—Insulin-like growth factor-binding protein 2; IGFBP-3—Insulin-like growth factor-binding protein 3; IGFBP-4—Insulin-like growth factor-binding protein 4; IL-1a—Interleukin-1 alpha; IL-1 b—Interleukin-1 beta; IL-2—Interleukin-2; IL-3—Interleukin-3; IL-4—Interleukin-4; IL-5—Interleukin-5; IL-6—Interleukin-6; IL-7—Interleukin-7; IL-8—Interleukin-8; IL-10—Interleukin-10; IL-12—Interleukin-12; IL-13—Interleukin-13; IL-15—Interleukin-15; IL-16—Interleukin-16; IP-10—Interferon gamma-induced protein; LIF—Leukemia inhibitory factor; LIGHT (TNFSF14)—Tumor necrosis factor ligand superfamily member 14; MCP-1—Monocyte chemoattractant protein 1; MCP-2—Monocyte chemoattractant protein 2; MCP-3—Monocyte chemoattractant protein 3; MCP-4—Monocyte chemoattractant protein 4; MCSF—Macrophage colony-stimulating factor 1; MDC—Macrophage-derived chemokine; MIF—Macrophage migration inhibitory factor; MIG—Monokine induced by interferon-gamma; MIP-1b—Macrophage inflammatory protein 1-beta; MIP-1d—Macrophage inflammatory protein 1-delta; MIP-3 a—Macrophage inflammatory protein 3 alpha; NAP-2—Neutrophil-activating peptide 2; NT-3—Neurotrophin-3; NT-4—Neurotrophin-4; PARC—Pulmonary and activation-regulated chemokine; PDGF-BB—Platelet-derived growth factor subunit B; PlGF—Placenta growth factor; RANTES (CCL5)—C-C motif chemokine 5; SCF—Stem cell factor; SDF-1—Stromal cell-derived factor 1; TARC(CCL17); TGF-β 1—Transforming growth factor beta-1; TGF-β 2—Transforming growth factor beta-2; TGF-β 3—Transforming growth factor beta-3; TIMP-1—Metalloproteinase inhibitor 1; TIMP-2—Metalloproteinase inhibitor 2; TNF-α —Tumor necrosis factor alpha; TNF-β —Tumor necrosis factor beta; VEGF—Vascular endothelial growth factor. The explanation of the cytokine abbreviations was obtained from the UniProt Database (http://www.uniprot.org/; accessed 10 October 2021).

## Data Availability

The data presented in this study are available in the article.
